# Red risks for a journey to the red planet: The highest priority human health risks for a mission to Mars

**DOI:** 10.1038/s41526-020-00124-6

**Published:** 2020-11-05

**Authors:** Zarana S. Patel, Tyson J. Brunstetter, William J. Tarver, Alexandra M. Whitmire, Sara R. Zwart, Scott M. Smith, Janice L. Huff

**Affiliations:** 1 KBR, Houston, TX USA; 2 NASA Lyndon B. Johnson Space Center, Houston, TX USA; 3 U.S. Navy, NASA Lyndon B. Johnson Space Center, Houston, TX USA; 4 University of Texas Medical Branch at Galveston, Galveston, TX USA; 5 NASA Langley Research Center, Hampton, VA USA

**Keywords:** Cardiovascular diseases, Eye diseases, Psychiatric disorders, Neurological disorders, Cancer

## Abstract

NASA’s plans for space exploration include a return to the Moon to stay—boots back on the lunar surface with an orbital outpost. This station will be a launch point for voyages to destinations further away in our solar system, including journeys to the red planet Mars. To ensure success of these missions, health and performance risks associated with the unique hazards of spaceflight must be adequately controlled. These hazards—space radiation, altered gravity fields, isolation and confinement, closed environments, and distance from Earth—are linked with over 30 human health risks as documented by NASA’s Human Research Program. The programmatic goal is to develop the tools and technologies to adequately mitigate, control, or accept these risks. The risks ranked as “red” have the highest priority based on both the likelihood of occurrence and the severity of their impact on human health, performance in mission, and long-term quality of life. These include: (1) space radiation health effects of cancer, cardiovascular disease, and cognitive decrements (2) Spaceflight-Associated Neuro-ocular Syndrome (3) behavioral health and performance decrements, and (4) inadequate food and nutrition. Evaluation of the hazards and risks in terms of the space exposome—the total sum of spaceflight and lifetime exposures and how they relate to genetics and determine the whole-body outcome—will provide a comprehensive picture of risk profiles for individual astronauts. In this review, we provide a primer on these “red” risks for the research community. The aim is to inform the development of studies and projects with high potential for generating both new knowledge and technologies to assist with mitigating multisystem risks to crew health during exploratory missions.

## Introduction

**Fig. 1:**
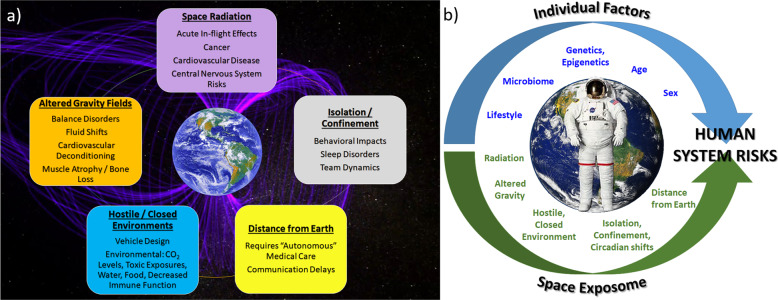
The five main hazards of spaceflight and the space exposome. **a** The key threats to human health and performance associated with spaceflight are radiation, altered gravity fields, hostile and closed environments, distance from Earth, and isolation and confinement. From these five hazards stem the health and performance risks studied by NASA’s Human Research Program. **b** The space exposome considers the summation of an individual’s environmental exposures and their interaction with individual factors such as age, sex, genomics, etc. - these interactions are ultimately responsible for risks to the human system. Images used in this figure are courtesy of NASA.

Spaceflight is a dangerous and demanding endeavor with unique hazards and technical challenges. Ensuring the overall safety of the crew—their physical and mental health and well-being—are vital for mission success. These are large challenges that are further amplified as exploration campaigns extend to greater distances into our solar system and for longer durations. The major health hazards of spaceflight include higher levels of damaging radiation, altered gravity fields, long periods of isolation and confinement, a closed and potentially hostile living environment, and the stress associated with being a long distance from mother Earth. Each of these threats is associated with its own set of physiological and performance risks to the crew (Fig. 1a) that must be adequately characterized and sufficiently mitigated. Crews do not experience these stressors independently, so it is important to also consider their combined impact on human physiology and performance. This “space exposome” is a unifying framework that reflects the interaction of all the environmental impacts on the human body (Fig. 1b) and, when combined with individual genetics, will shape the outcomes of space travel on the human system^1,2^.

**Fig. 2:**
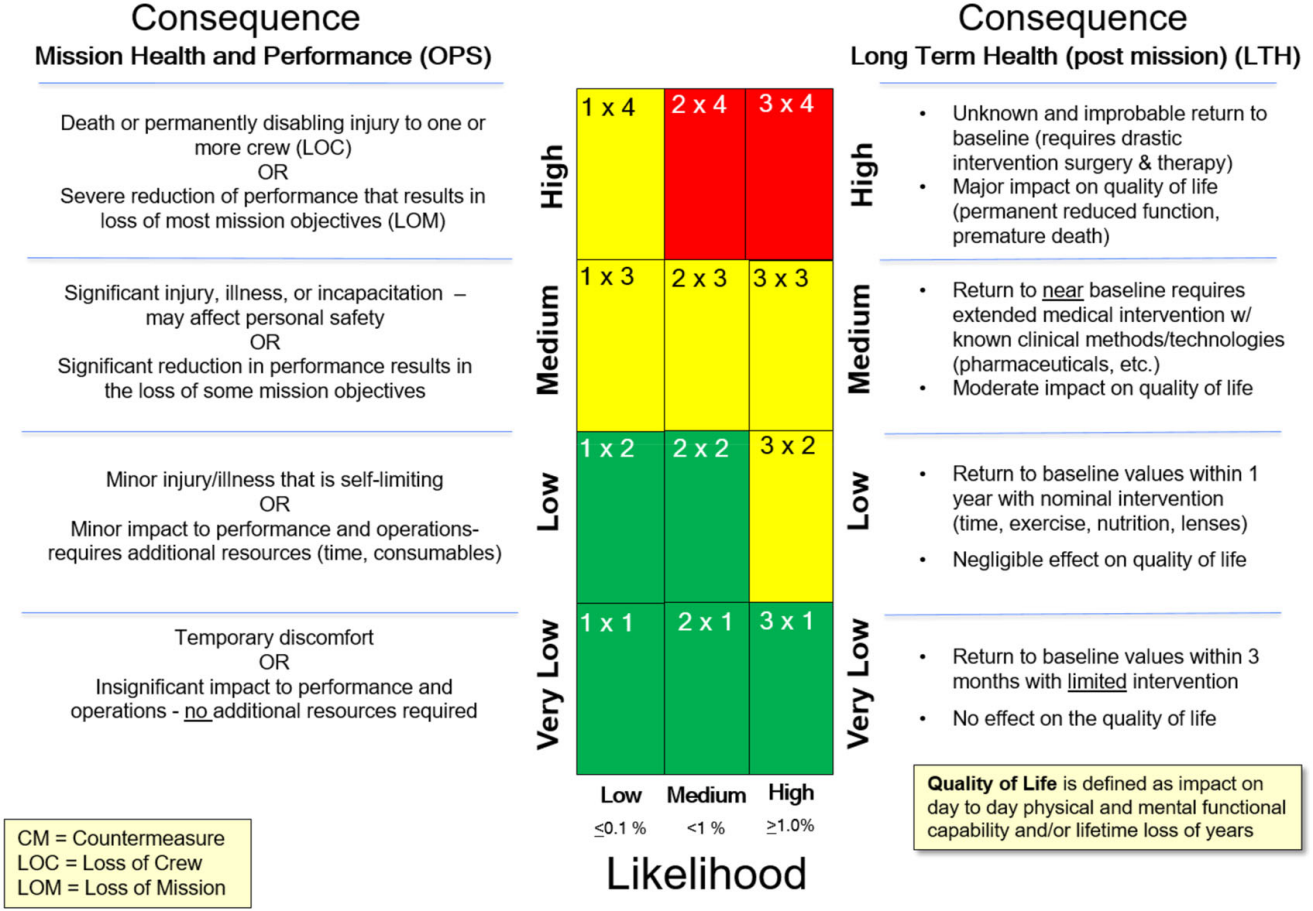
NASA human system risks—likelihood and consequence rating scale. NASA uses an evidence-based approach to assess likelihood and consequence for each documented human system risk. The matrix used for classifying and prioritizing human system risks has two sets of consequences—the left side shows consequences for in-mission risks while the right side is used to evaluate long-term health consequences (Romero and Francisco)^4^.

The NASA Human Research Program (HRP) aims to develop and provide the knowledge base, technologies, and countermeasure strategies that will permit safe and successful human spaceflight. With agency resources and planning directed toward extended missions both within low Earth orbit (LEO) and outside LEO (including cis-lunar space, lunar surface operations, a lunar outpost, and exploration of Mars)^3^, HRP research and development efforts are focused on mitigation of over 30 categories of health risks relevant to these missions. The HRP’s current research strategy, portfolio, and evidence base are described in the HRP Integrated Research Plan (IRP) and are available online in the Human Research Roadmap, a managed tool used to convey these plans (https://humanresearchroadmap.nasa.gov/). To determine research priorities, NASA uses an evidence-based risk approach to assess the likelihood and consequence (LxC), which gauges the level of each risk for a set of standard design reference missions (Fig. 2)^4^. Risks are assigned a rating for their potential to impact in-mission crew health and performance and for their potential to impact long-term health outcomes and quality of life. “Red” risks are those that are considered the highest priority due to their greatest likelihood of occurrence and their association with the most significant risks to crew health and performance for a given design reference mission (DRM). Risks rated “yellow” are considered medium level risks and are either accepted due to a very low probability of occurrence, require in-mission monitoring to be accepted, or require refinement of standards or mitigation strategies in order to be accepted. Risks rated “green” are considered sufficiently controlled either due to lower likelihood and consequence or because the current knowledge base provides sufficient mitigation strategies to control the risk to an acceptable level for that DRM. Milestones and planned program deliverables intended to move a risk rating to an acceptable, controlled level are detailed in a format known as the path to risk reduction (PRR) and are developed for each of the identified risks. The most recent IRP and PRR documents are useful resources for investigators during the development of relevant research approaches and proposals intended for submission to NASA HRP research announcements (https://humanresearchroadmap.nasa.gov/Documents/IRP_Rev-Current.pdf).

This work reviews HRP-defined high priority “red” risks for crew health on exploration missions: (1) space radiation health effects that include cancer, cardiovascular disease, and cognitive decrements (2) Spaceflight-Associated Neuro-ocular Syndrome (3) behavioral health and performance decrements, and (4) inadequate food and nutrition. The approaches used to address these risks are described with the aim of informing potential NASA proposers on the challenges and high priority risks to crew health and performance present in the spaceflight environment. This should serve as a primer to help individual proposers develop projects with high potential for generating both new knowledge and technology to assist with mitigating risks to crew health during exploratory missions.

## Space radiation health risks

Outside of the Earth’s protective magnetosphere, crews are exposed to pervasive, low dose-rate galactic cosmic rays (GCR) and to intermittent solar particle events (SPEs)^5^. Exposures from GCR are from high charge (Z) and energy (HZE) ions, high-energy protons, and secondary protons, neutrons, and fragments produced by interactions with spacecraft shielding and human tissues. The main components of an SPE are low-to-medium energy protons. In LEO, the exposures are from GCR modulated by the Earth’s magnetic field and from trapped protons in the South Atlantic Anomaly. The absorbed doses for crews on the International Space Station (ISS) on 6- to 12-month missions range from ~30 to 120 mGy. Outside of LEO, without the protection offered by the Earth’s magnetosphere, absorbed radiation doses will be significantly higher. Estimates for a 1 year stay on the lunar surface range from 100 to 120 mGy, and 300 to 450 mGy for an ~3-year Mars mission (transit and surface stay)^6^. The exact dose a crewmember will receive is highly dependent on exact parameters of a given mission, such as detailed vehicle and habitat designs, and mission location and duration^7^. Time in the solar cycle is also a large factor contributing to crew exposure, with highest GCR exposure occurring during periods of minimum solar activity. The lowest GCR exposures occur during periods of maximum solar activity when the heightened magnetic activity of the Sun diverts some cosmic rays; however, during maximum solar activity, the probability of an SPE is higher^8,9^. SPEs, which vary in the magnitude and frequency, will obviously also contribute to total mission doses so it is important to note that total mission exposures are only estimates. Further information on the space radiation environment that astronauts will experience is discussed in Simonsen et al.^5^ and Durante and Cucinotta^10^.

**Fig. 3:**
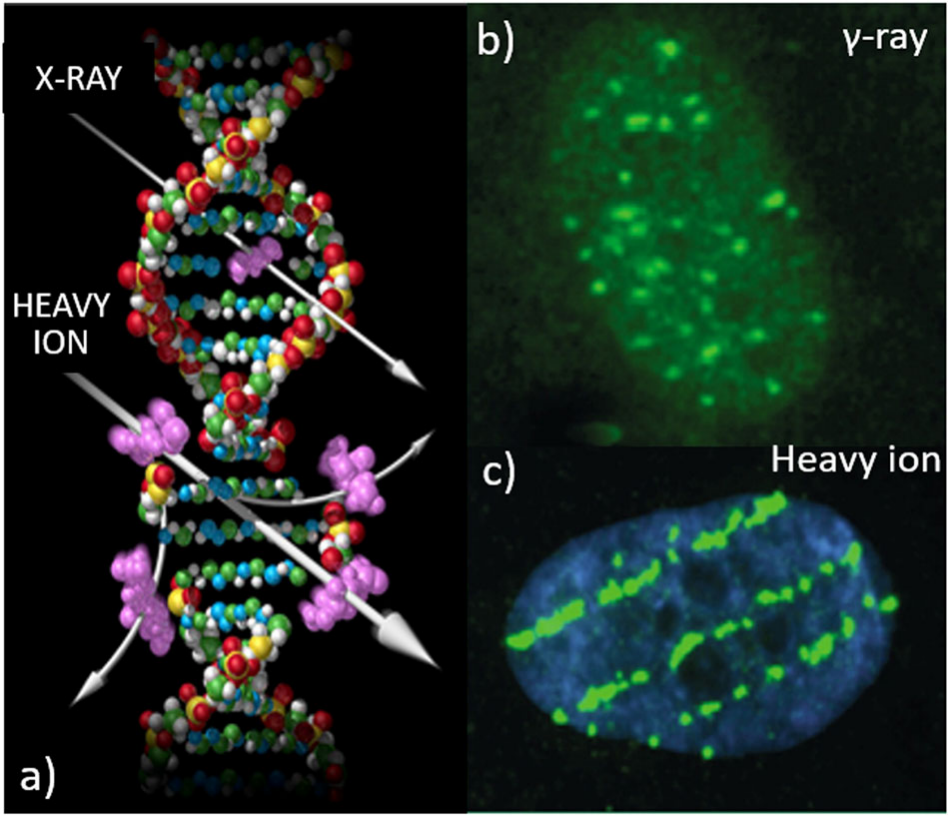
Galactic cosmic rays are qualitatively different from X-rays or gamma-rays. **a** HZE ions produce dense ionization along the particle track as they traverse a tissue and impart distinct patterns of DNA damage compared to terrestrial radiation such as X-rays. γH2AX foci (green) illuminate distinct patterns of DNA double-strand breaks in nuclei of human fibroblast cells after exposure to **b** gamma-rays, with diffuse damage, and **c** HZE ions with single tracks. Image credits: NASA (**a**) and Cucinotta and Durante^97^ (**b** and **c**).

An important consideration for risk assessment is that the types of radiation encountered in space are very different from the types of radiation exposure we are familiar with here on Earth. HZE ions, although a small fraction of the overall GCR spectrum compared to protons, are more biologically damaging. They differ from terrestrial forms of radiation, such as X-rays and gamma-rays, in both the amount (dose) of exposure as well as in the patterns of DNA double-strand breaks and oxidative damage that they impart as they traverse through tissue and cells (Fig. 3)^5^. The highly energetic HZE particles produce complex DNA lesions with clustered double-stranded and single-stranded DNA breaks that are difficult to repair. This damage leads to distinct cellular behavior and intracellular signaling patterns that may be associated with altered disease outcomes compared to those for terrestrial sources of radiation^11–13^. As an example, persistently high levels of oxidative damage are observed in the intestine from mice examined 1 year after exposure to ^56^Fe-ion radiation compared to gamma radiation and unirradiated controls^14,15^. The higher levels of residual oxidative damage in HZE ion-irradiated tissue is significant because of the association of oxidative stress and damage with the etiology of many human diseases, including cancer, cardiovascular and late neurodegenerative disorders. These types of alterations are believed to contribute to the higher biological effectiveness of HZE particles^10,11^.

Within the HRP, the Space Radiation Element (SRE) has developed a research strategy involving both vertical translation and horizontal integration, as well as products focused on mitigating space radiation risks across all phases of a mission. Vertical translation involves the integration of benchtop research with preclinical studies and clinical data. Horizontal integration involves a multidisciplinary approach that includes a range of expertize from physicians to clinicians, epidemiologists to computational modelers^16^. The suite of tools includes computational models of the space radiation environment, mission design tools, models for risk projection, and tools and technologies for accurate simulation of the space radiation environment for radiobiology investigations. Ongoing research is focused on radiation quality, age, sex, and healthy worker effects, medical countermeasures to reduce or eliminate space radiation health risks, understanding the complex nature of individual sensitivity, identification and validation of biomarkers (translational, surrogate, predictive, etc.) and integration of personalized risk assessment and mitigation approaches. Owing to the lack of human data for heavy ion exposure on Earth and the complications of obtaining reliable data for space radiation health effects from flight studies, SRE conducts research at the NASA Space Radiation Laboratory (NSRL) at Brookhaven National Laboratory. The NSRL is a ground-based analog for space radiation, where a beamline and associated experimental facilities are dedicated to the radiobiology and physics of a range of ions from proton and helium ions to the typical GCR ions such as carbon, silicon, titanium, oxygen, and iron^5,17,18^.

### Radiation carcinogenesis

Central evidence for association between radiation exposure and the development of cancer and other non-cancer health effects comes from epidemiological studies of humans exposed to radiation^19–22^. Scaling factors are used by NASA and other space agencies in the analysis of cancer (and other risks) to account for differences between terrestrial radiation exposures and cosmic radiation exposures^23^. The risk of radiation carcinogenesis is considered a “red” risk for exploration-class missions due to both the high likelihood of occurrence, as well as the high potential for detrimental impact on both quality of life and disease-free survival post flight. The major cancers of concern are epithelial in origin (particularly cancers of the lung, breast, stomach, colon, and bladder), as well as leukemias (https://humanresearchroadmap.nasa.gov/Evidence/reports/Cancer.pdf). Owing to the lack of human epidemiology directly relevant to the types of radiation found in space, current research utilizes a translational approach that incorporates rodent and advanced human cell-based model systems exposed to space radiation simulants along with comparison of molecular pathways across these systems to the human.

**Fig. 4:**
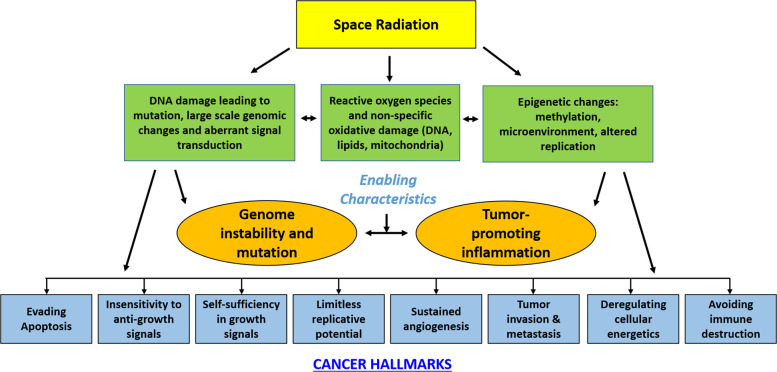
The hallmarks and emerging hallmarks of cancer. Shown are the enabling characteristics and possible mechanisms of radiation damage that lead to these changes observed in all human tumors. (Adapted from Hanahanand Weinberg)^24^.

A key question that impacts risk assessment and mitigation is how HZE tumors compare to either radiogenic tumors induced by ground-based radiation or spontaneous tumors. As a unifying concept, NASA studies have sought to examine how space radiation exposure modifies the key genetic and epigenetic modifications noted as the hallmarks of cancer (Fig. 4)^24–27^. This approach provides data for development of translational scaling factors (relative biological effectiveness values, quality factors, dose-rate effectiveness factor) to relate the biological effects of space radiation to effects from similar exposures to ground-based gamma- and X-rays and extrapolation of results to large human epidemiology cohorts. It also supports acquisition of mechanistic information required for successful identification and implementation of medical countermeasure strategies to lower this risk to an acceptable posture for space exploration, and it is relevant for the future development of biologically based dose-response models and integrated systems biology approaches^25^. Cancer is a long-term health risk and although it is rated as “red”, most research in this area is currently delayed, as HRP research priorities focus on in-mission risks.

### Risk of cardiovascular disease and other degenerative tissue effects from radiation exposure and secondary spaceflight stressors

**Fig. 5:**
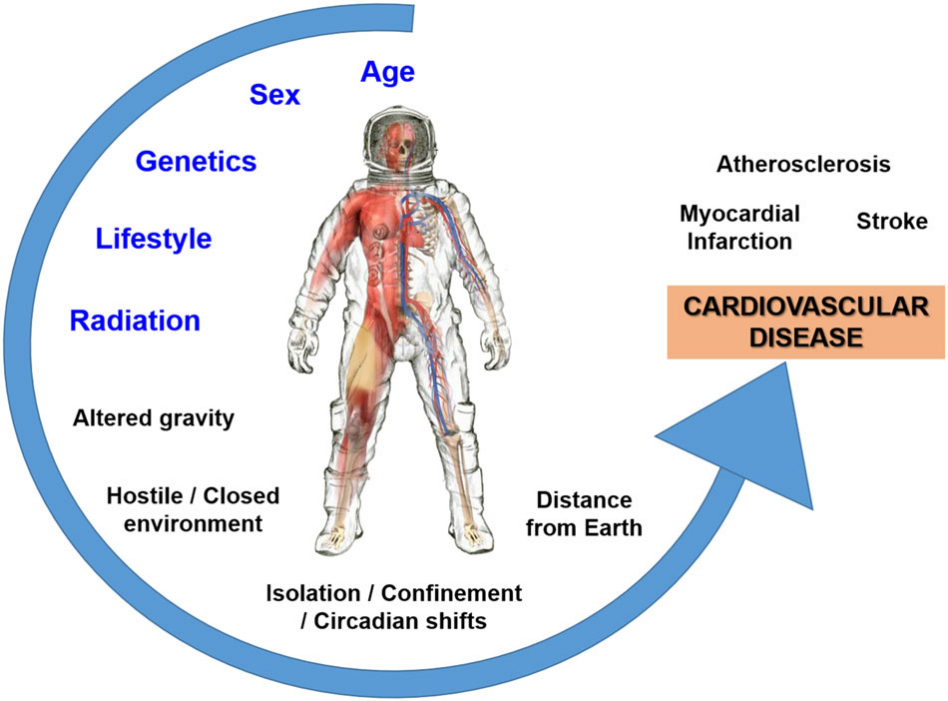
Cardiovascular disease is a human systems risk. In blue are the known risk factors for CVD and in black are the other spaceflight stressors that may also contribute to disease development. Image used in this figure is courtesy of NASA.

A large number of degenerative tissue (non-cancer) adverse health outcomes are associated with terrestrial radiation exposure, including cardiovascular and cerebrovascular diseases, cataracts, digestive and endocrine disorders, immune system decrements, and respiratory dysfunction (https://humanresearchroadmap.nasa.gov/Evidence/other/Degen.pdf). For cardiovascular disease (CVD), a majority of the evidence comes from radiotherapy cohorts receiving high-dose mediastinal exposures that are associated with an increased risk for heart attack and stroke^28^. Recent evidence shows risk at lower doses (<0.5 Gy), with an estimated latency of 10 years or more^29–31^. For a Mars mission, preliminary estimates suggest that circulatory disease risk may increase the risk of exposure induced death by ~40% compared to cancer alone^32^. NASA is also concerned about in-flight risks to the cardiovascular system (https://humanresearchroadmap.nasa.gov/Evidence/other/Arrhythmia.pdf), when considering the combined effects of radiation exposure and other spaceflight hazards (Fig. 5)^33^. The Space Radiation Element is focused on accumulating data specific to the space radiation environment to characterize and quantify the magnitude of the degenerative disease risks. The current efforts are on establishing dose thresholds, understanding the impact of dose-rate and radiation quality effects, uncovering mechanisms and pathways of radiation-associated cardiovascular and cerebrovascular diseases, and subsequent risk modeling for astronauts. Uncovering the mechanistic underpinnings governing disease processes supports the development of specific diagnostic and therapeutic approaches, is a necessary step in the translation of insights from animal models to humans, and is the basis of personalized medicine approaches.

This information will provide a means to reduce the uncertainty in current permissible exposure limits (PELs), quantify the impact to disease-free survival years, and determine if additional protection or mitigation strategies are required. The research portfolio includes evaluation of current clinical standard-of-care biomarkers for their relevance as surrogate endpoints for radiation-induced disease outcomes. Studies are also addressing the possible role of chronic inflammation and increased oxidative stress in the etiology of radiation-induced CVD, as well as identification of key events in disease pathways, like endothelial dysfunction, that will guide the most effective medical countermeasures. Products include validated space radiation PELs, models to quantify the risk of CVD for the astronaut cohort, and countermeasures and evidence to inform development of appropriate recommendations to clinical guidelines for diagnosis and mitigation of this risk.

Elucidating the role that radiation plays in degenerative disease risks is problematic because multiple factors, including lifestyle and genetic influences, are believed to play a major role in the etiology of these diseases. This confounds epidemiological analyses, making it difficult to detect significant differences from background disease without a large study population^34^. This issue is especially significant in astronaut cohorts because those studies have small sample sizes^35^. There is also a general lack of experimental data that specifically addresses the role of radiation at low, space-relevant doses^36^. Selection of experimental models needs to be carefully considered and planned to ensure that the cardiovascular disease mechanisms and study endpoints are clinically relevant and translatable to humans^37,38^. Combined approaches using data from wildtype and genetically modified animal models with accelerated disease development will likely be necessary to elucidate mechanisms and generate the body of knowledge required for development of accurate permissible exposure limits, risk assessment models, and to develop effective mitigation approaches.

### Risk of acute (in-flight) and late CNS effects from space radiation exposure

**Fig. 6:**
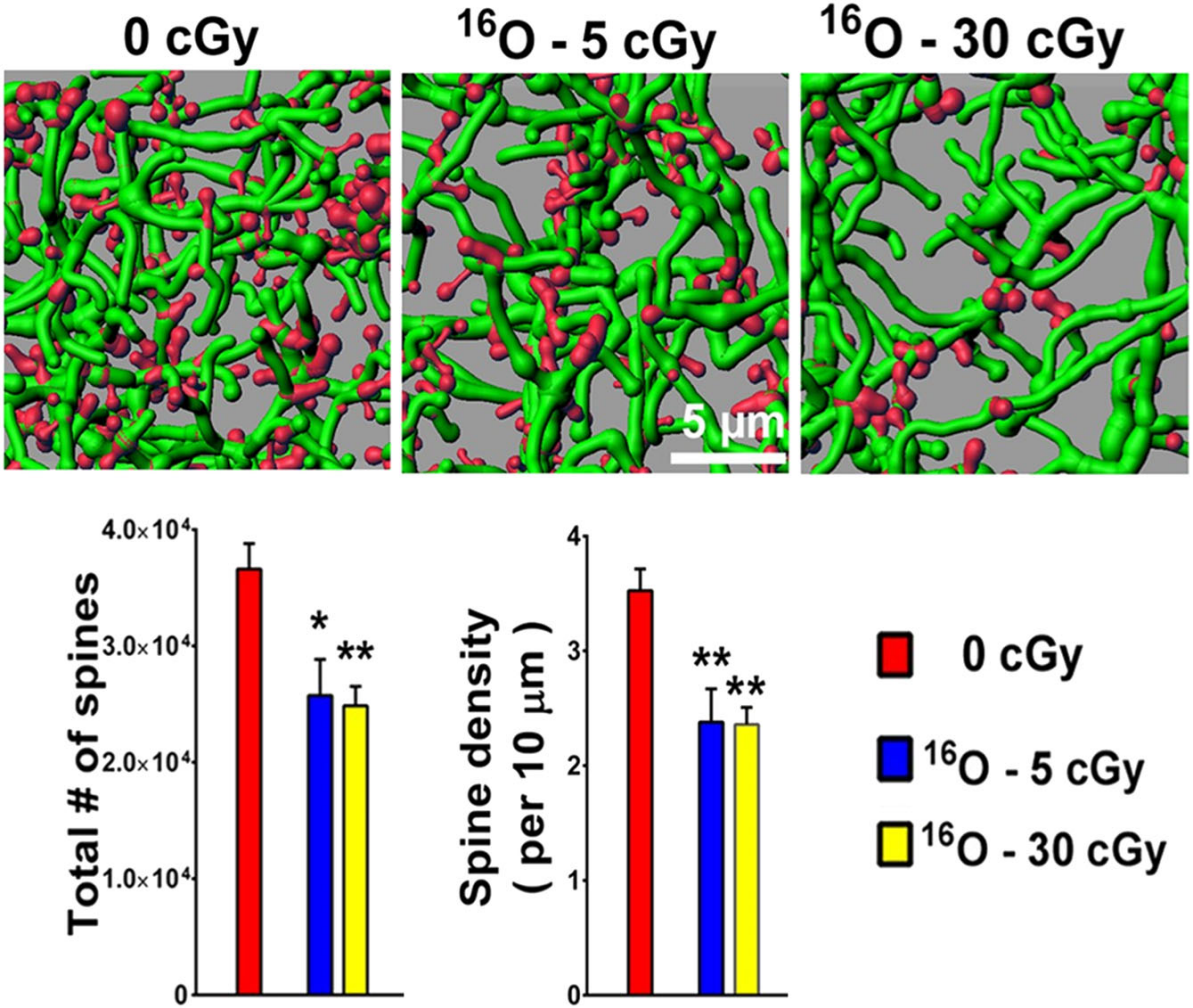
Reduced dendritic spine density in the rodent medial prefrontal cortex 15 weeks following exposure to cosmic radiation. Representative digital images of 3D reconstructed dendritic segments (green) containing spines (red) in unirradiated (0 cGy) and irradiated (5 and 30 cGy) mice brains. Multiple comparisons show that total spine numbers (left bar chart) and spine density (right bar chart) are significantly reduced after exposure to 5 or 30 cGy of ^16^O particles. Data are expressed as mean ± SEM. *P < 0.05, **P < 0.01 versus control; ANOVA. Adapted from Parihar et al.^39^. Permission to reproduce open-source figure per the Creative Commons Attribution 4.0 International License. https://creativecommons.org/licenses/by/4.0.

The possibility of acute (in-flight) and late risks to the central nervous system (CNS) from GCR and SPEs are concerns for human exploration of space (https://humanresearchroadmap.nasa.gov/Evidence/reports/CNS.pdf). Acute CNS risks may include altered neurocognitive function, impaired motor function, and neurobehavioral changes, all of which may affect human health and performance during a mission. Late CNS risks may include neurological disorders such as Alzheimer’s disease, dementia, or accelerated aging. Detrimental CNS changes from radiation exposure are observed in humans treated with high doses of gamma-rays or proton beams and are supported by a large body of experimental evidence showing neurocognitive and behavioral effects in animal models exposed to lower doses of HZE ions. Rodent studies conducted with HZE ions at low, mission-relevant doses and time frames show a variety of structural and functional alterations to neurons and neural circuits with associated performance deficits^39–44^. Fig. 6 shows an example of changes in dendritic spine density following HZE ion radiation. However, the significance and relationship of these results to adverse outcomes in astronauts is unclear, as similar decrements are not seen with comparable doses of terrestrial radiation. Therefore, scaling to human epidemiology data, as is done for cancer and cardiovascular disease, is not possible. It is also important to note that to date, no radiation-associated clinically significant operational or long-term deficits have been identified in astronauts receiving similar doses via long-duration ISS missions. It is clear that further development of standardized translational models, research paradigms, and appropriate scaling approaches are required to determine significance in humans^45,46^. In addition, elucidation of how space radiation interacts with other mission hazards to impact neurocognitive and behavioral health and performance is critical to defining appropriate PELs and countermeasure strategies. The current research approach is a combined effort of SRE, the human factors and behavioral performance element, and the human health countermeasures element in support of an integrated CBS (CNS/behavioral medicine/sensorimotor) plan (https://humanresearchroadmap.nasa.gov/Risks/risk.aspx?i=99). Further information on this risk area is presented below in the Behavioral Health and Performance section and can also be found at the Human Research Roadmap.

To summarize, the health risks posed by the omnipresent exposure to space radiation are significant and include the “red” risks of cancer, cardiovascular diseases, and cognitive and behavioral decrements. While research on the late health risk of cancer is currently delayed, research on the in-flight effects of radiation on the cardiovascular system and CNS within the context of the space exposome are considered the highest priority and are the focus of investigations. Major knowledge gaps include the effects of radiation quality, dose-rate, and translation from animal models to human systems and evaluation of the requirement for medical countermeasure approaches to reduce the risk.

## Spaceflight-Associated Neuro-ocular Syndrome

The Risk of Spaceflight-Associated Neuro-ocular Syndrome (SANS), originally termed the Risk of Vision Impairment Intracranial Pressure (VIIP), was first discovered about 15 years ago. VIIP was the original name used because the syndrome most noticeably affects a crewmember’s eyes and vision, and its signs can appear like those of the terrestrial condition idiopathic intracranial hypertension (IIH; which is due to increased intracranial pressure). Over time, it was realized that the VIIP name required an update. Most notably, SANS is not associated with the classic symptoms of increased intracranial pressure in IIH (e.g., severe headaches, transient vision obscurations, double vision, pulsatile tinnitus), and it has never induced vision changes that meet the definition of vision impairment, as defined by the National Eye Institute. In 2017, VIIP was renamed to SANS, a term that welcomes additional pathogenesis theories and serves as a reminder that this syndrome could affect the CNS well beyond the retina and optic nerve.

**Fig. 7:**
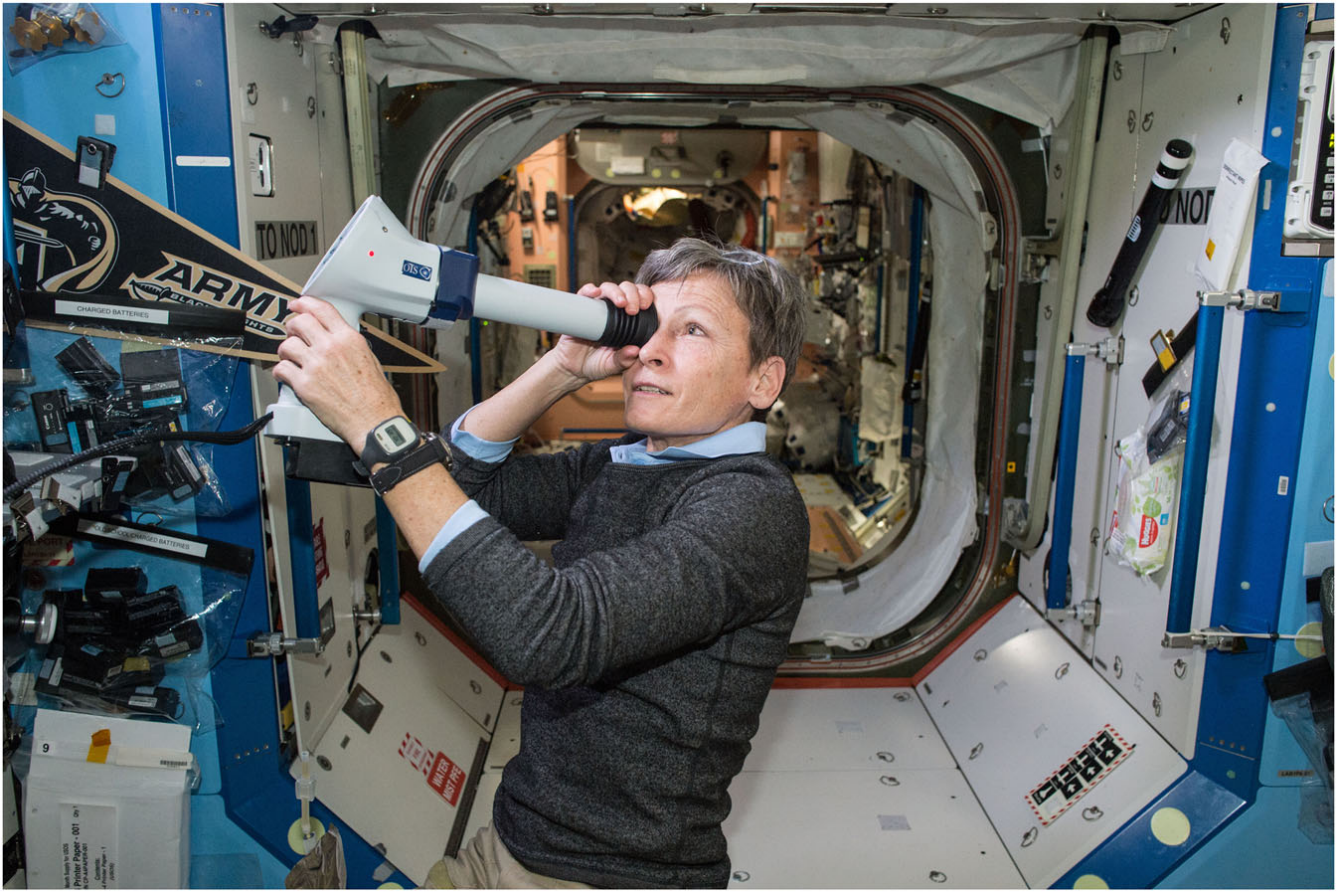
Onboard the ISS, NASA astronaut Peggy Whitson collects images of the back of the eye during a routine screening check. Image courtesy of NASA.

SANS presents with an array of signs, as documented in the HRP Evidence Report (https://humanresearchroadmap.nasa.gov/evidence/reports/SANS.pdf). Primarily, these include edema (swelling) of the optic disc and retinal nerve fiber layer (RNFL), chorioretinal folds (wrinkles in the retina), globe flattening, and refractive error shifts^47^. Flight duration is thought to play a role in the pathogenesis of SANS, as nearly all cases have been diagnosed during or immediately after long-duration spaceflight (i.e., missions of 30 days duration or longer), although signs have been discovered as early as mission day 10^48^. Because of SANS, ocular data are nominally collected during ISS missions. For most ISS crewmembers, this testing includes optical coherence tomography (OCT), retinal imaging, visual acuity, a vision symptom questionnaire, Amsler grid, and ocular ultrasound (Fig. 7).

From a short-term perspective (e.g., a 6-month ISS deployment), SANS presents four main risks to crewmembers and their mission: optic disc edema (ODE), chorioretinal folds, shifts in refractive error, and globe flattening^49^. Approximately 69% of the US crewmembers on the ISS experience a > 20 µm increase in peripapillary retinal thickness in at least one eye, indicating the presence of ODE. With significant levels of ODE, a crewmember can experience an enlargement of his/her blind spots and a corresponding loss in visual function. To date, blind spots are uncommon and have not had an impact on mission performance.

If chorioretinal folds are severe enough and located near the fovea (the retina associated with central vision), a crewmember may experience visual distortions or reduced visual acuity that cannot be corrected with glasses or contact lenses, as noted in the SANS Evidence Report. Despite a prevalence of 15–20% in long-duration crewmembers, chorioretinal folds have not yet impacted astronauts’ visual performance during or after a mission. An on-orbit shift in refractive error is due to a shortening of the eye’s axial length (distance between the cornea and the fovea), and it occurs in about 16% of crewmembers during long-duration spaceflight. This risk is mitigated by providing deploying crewmembers with several pairs of “Space Anticipation Glasses” (or contact lenses) of varying power. On-orbit, the crewmember can then select the appropriate lenses to restore best-corrected visual acuity. Approximately 29% of long-duration crewmembers experience a posterior eyeball flattening, which is typically centered around the insertion of the optic nerve into the globe. Globe flattening can induce chorioretinal folds and shifts in refractive error, posing the respective risks described above.

From a longer-term perspective, SANS presents two main risks to crewmembers: ODE and chorioretinal folds. It is unknown if a multi-year spaceflight (e.g., a Mars mission) will be associated with a higher prevalence, duration, and/or severity of ODE compared to what has been experienced onboard the ISS. Since the retina and optic nerve are part of the CNS, if ODE is severe enough, the crewmember risks a permanent loss of optic nerve and RNFL tissue and thus, a permanent loss of visual function. It should be stressed that no SANS-related permanent loss of visual function has yet been discovered in any astronauts.

For choroidal folds, improvement generally occurs post-flight in affected crewmembers; however, significant folds can persist for 10 or more years after long-duration missions. Using MultiColor Imaging and autofluorescence capabilities of the latest OCT device, it was discovered recently that one crewmember’s longstanding (>5 years) post-flight choroidal folds have induced disruption to its overlying retinal pigment epithelium (RPE)^50^. The RPE is a monolayer of pigmented cells located between the vascular-rich choroid and the photoreceptor outer segments. This layer forms the posterior blood-brain barrier for the retina and is essential for maintaining the health of the posterior retina via the transport of nutrients and fluids, among other key functions. If the RPE is damaged, it could potentially lead to a degeneration of the local retina and progress to vision impairment.

**Fig. 8:**
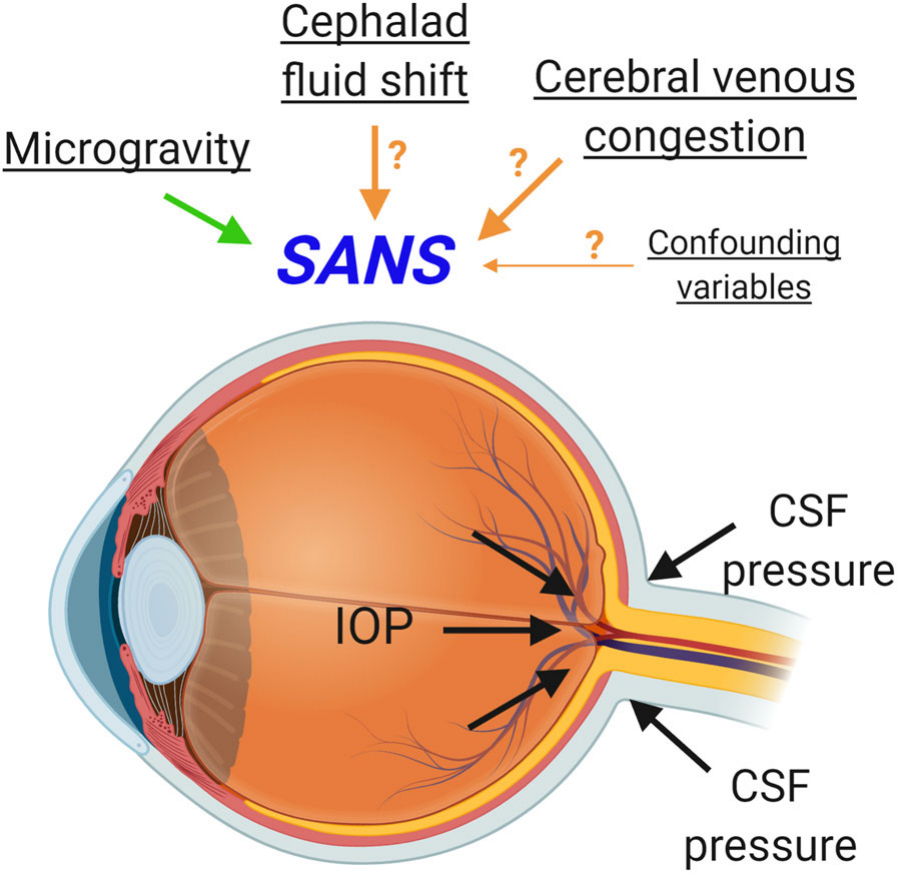
Potential pathways for SANS. Image created with BioRender.com.

Recent long-duration head-down tilt studies have shown potential for recreating SANS signs in terrestrial cohorts^51^. However, SANS is considered a pathology unique to spaceflight. In microgravity, fluid within the body is free to redistribute uniformly. This means that much of the fluid that normally pools in a person’s feet and legs due to gravity can transfer upward towards the head and cause a general congestion of the cerebral venous system. The central pathogenesis theories of SANS are based on these facts, but the actual cause(s) and pathophysiology of SANS are yet unknown^49^. The most publicized theory for SANS has been that cerebral spinal fluid outflow might be impeded, causing an overall increase in intracranial pressure (ICP)^47,52^. Other potential mechanisms (see Fig. 8) include cerebral venous congestion or altered folate-dependent 1-carbon metabolism via a cascade of mechanisms that may ultimately increase ICP or affect the response of the eye to fluid shifts^53,54^. Potential confounding variables for SANS pathogenesis include resistive exercise, high-sodium dietary intake, and high carbon dioxide levels.

Discovering patterns and trends in the SANS population has been difficult due to the relatively low number of crewmembers who have completed long-duration spaceflight. This is especially true for female astronauts. However, there is now enough evidence to state—emphatically—that SANS is not a male-only syndrome. OCT has been utilized onboard the ISS since late 2013, and it has revolutionized NASA’s ability to objectively detect and monitor SANS and build a high-resolution database of retinal and optic nerve head images. Through this technology, it has been recently discovered that that a majority of long-duration astronauts (including females) present with some level of ODE and engorgement of the choroidal vasculature^48,55^. The trends and patterns of these ocular anatomical changes may hold the key to deciphering the pathophysiology of SANS^48,55^.

Beginning in 2009 in response to SANS, all NASA crewmembers receive pre- and post-flight 3 Tesla magnetic resonance imaging of the brain and orbits. Based on these images, there is growing evidence that brain structural changes also occur during long-duration spaceflight. Most notably, a 10.7–14.6% ventricular enlargement (i.e., approximately a 2–3 ml increase) has been detected in astronauts and cosmonauts by multiple investigators^56–59^. On-orbit and post-flight cognitive testing have not revealed any systemic cognitive decrements associated with these anatomical changes. Moreover, additional research is required to determine if spaceflight-associated brain structural changes are related to ocular structural changes (i.e., SANS) or if the two are initiated by a common cause. Thus, until a relationship is established, SANS will be defined by ocular signs.

Future SANS medical operations, research, and surveillance will focus on: 1) determining the pathogenesis of the syndrome, 2) developing small-footprint diagnostic devices for expeditionary spaceflight, 3) establishing effective countermeasures, 4) monitoring for any long-term health consequences, and 5) discovering what factors make certain individuals more susceptible to developing the syndrome.

In summary, SANS is a top risk and priority to NASA and HRP. The primary SANS-related risk is ODE, due to the possibility of permanent vision impairment; however, choroidal folds also present a short- and long-term risk to astronaut vision. Shifts in refractive error are relatively common in long-duration missions, but crewmembers do not experience a loss of visual acuity if adequate correction is available. SANS affects female astronauts, not just males, although it is not yet known if SANS prevalence is equal between the sexes. There are no terrestrial pathologies identical to SANS, including IIH. Long-duration spaceflight is also associated with brain anatomical changes; however, it is not yet known whether these changes are related to SANS. Finally, the pathogenesis of SANS remains elusive; however, the main theories are related to increased intracranial pressure, ocular venous congestion, and individual anatomical/genetic variability.

## Behavioral health and performance

The Risk of Adverse Cognitive and Behavioral Conditions and Psychiatric Disorders (BMed) focuses on characterizing and mitigating potential decrements in performance and psychological health resulting from multiple spaceflight hazards, including isolation and distance from earth. Spaceflight radiation is also recognized as contributing factor, particularly relative to a deep space planetary mission. The potential of additive or synergistic effects on the CNS resulting from simultaneous exposures to radiation, isolation and confinement, and prolonged weightlessness, is also of emerging concern (https://humanresearchroadmap.nasa.gov/Risks/risk.aspx?i=99).

The official risk statement in the BMed Evidence Report notes, “*given the extended duration of future missions and the isolated, confined and extreme environments, there is a possibility that (a) adverse cognitive or behavioral conditions will occur affecting crew health and performance; and (b) mental disorders could develop should adverse behavioral conditions be undetected and unmitigated*” (https://humanresearchroadmap.nasa.gov/Evidence/reports/BMED.pdf). Primary outcomes for this risk include decrements in cognitive function, operational performance, and psychological and behavioral states, with the development of psychiatric disorders representing the least likely but one of the most consequential outcomes crew could experience in extended spaceflight. BMed is considered a “red” risk for planetary missions, given the long-duration of isolation, extended confinement, and exposure to additional stressors, including increased radiation exposure. The Human Factors and Behavioral Performance Element within HRP utilizes a research strategy that incorporates flight studies on astronauts, research in astronaut-like individuals and teams in ground analogs, and works with the Space Radiation Element to use animal models supporting research on combined spaceflight stressors.

While astronauts successfully accomplish their mission objectives and report very positive experiences living and working in space, some anecdotal accounts from current and past astronauts suggest that psychological adaptation in the long-duration spaceflight environment can be challenging. However, clinically significant operational decrements have not been documented to date, as noted in the BMed Evidence Report. Discrete events that have been documented include accounts of adverse responses to workload by Shuttle payload specialists, and descriptions of ‘hostile’ and ‘irritable’ crew in the 84-day Skylab 4 mission, as well as symptoms of depression reported on Mir by 2 of the 7 NASA astronauts.

Currently, potential stressors affiliated with missions to the ISS include extended periods of high workload and/or schedule shifting, physiological adaptation including fluid shifts caused by weightlessness and possibly, exposure to other environmental factors such as elevated carbon dioxide (see the BMed Evidence Report). While still physically isolated from home, the presence of the ISS in LEO facilitates a robust ground behavioral health and performance support team who offer services such as bi-weekly private psychological conferences and regular delivery of novel goods and surprises from home in crew care packages. Coupled with the relatively ample volume in the ISS, near-constant real-time communication with Earth, new crewmembers rotating periodically throughout missions, and relatively low levels of radiation exposure, —it is expected that behavioral challenges experienced today do not represent those that future crews will face during exploration missions.

**Fig. 9:**
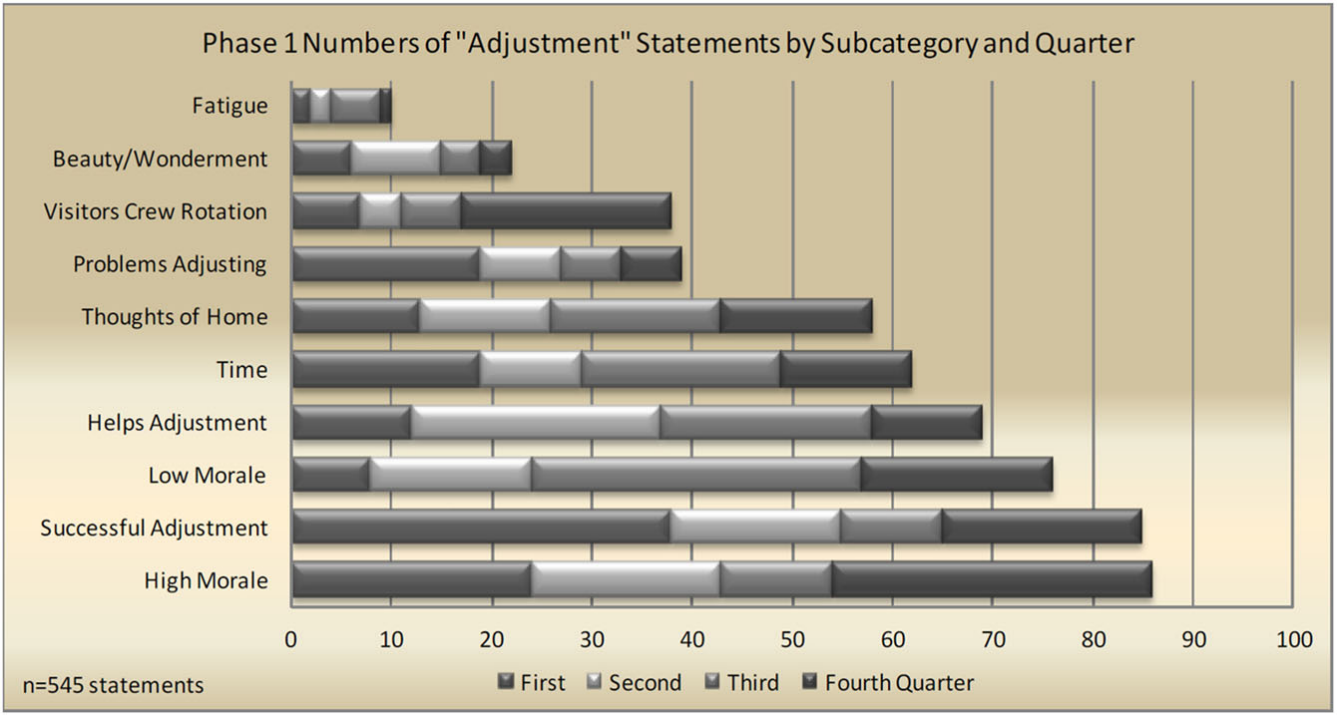
The “Journals” study of in-flight behavioral responses. Example bar graph showing distribution of journal entries related to general adjustment to the spaceflight enivronment during each quarter of an ISS mission^61^.

Nevertheless, the few completed behavioral studies on the ISS suggest that subjective perceptions of stress increase over time for some crewmembers, as shown by an in-flight study collecting subjective ratings of well-being and objective measures of fatigue^60^. Notably, it was found that astronaut ratings of sleep quality and sleep duration (also measured through visual analog scales) were found to be inversely related to ratings of stress. Another in-flight investigation seeking to characterize behavioral responses to spaceflight is the “Journals” study by Stuster^61^. This investigation provided a systematic approach to examining a rich set of qualitative data by evaluating astronaut journal entries for temporal patterns of across different behavioral states over the course of a mission (Fig. 9). Based on findings, some categories suggest temporal patterns while other categories of outcomes do not suggest a pattern relative to time, which may be due to no temporal relationship between outcomes and time, and/or various contextual factors within missions that negate the presence of such a relationship (e.g., visiting crew). An overall assessment by Stuster of negative comments relative to positive comments over time suggests evidence of a third quarter phenomenon in Adjustment alone, a category which reflects individual morale^61^.

Other in-flight investigations support and expand upon contributors to increased stress on-orbit, including studies documenting reductions in sleep duration^62,63^ and evaluation of crew responses to habitability and human factors during spaceflight^64^. While no studies have assessed potentially relevant mechanisms for behavioral or other reported symptoms, a recently completed investigation suggests neurostructural changes may be occurring in the spaceflight environment^56^. Magnetic resonance imaging scans were conducted on astronauts pre- and post-flight on both long-duration missions to the ISS or short-duration Shuttle missions. Assessments from a subgroup of participants (*n* = 12) showed a slight upward shift of the brain after all long-duration flights but not after short-duration flights (*n* = 6), and they also showed narrowing of cerebral spinal fluid spaces at the vertex after all long-duration flights (*n* = 6) and in 1 of 6 crew after short-duration flights. A retrospective analysis of free water volume in the frontal, temporal, and occipital lobes before versus after spaceflight suggests alterations in free water distribution^65^. Whether there is a functionally relevant outcome as a result of such changes remains to be determined. Hence, while certain aspects of the spaceflight environment have been shown to increase some behavioral responses (e.g., reduced sleep owing to workload), the direct role of spaceflight-specific factors (such as fluid shifts and weightlessness) on behavioral outcomes or functional performance has not yet been established.

**Fig. 10:**
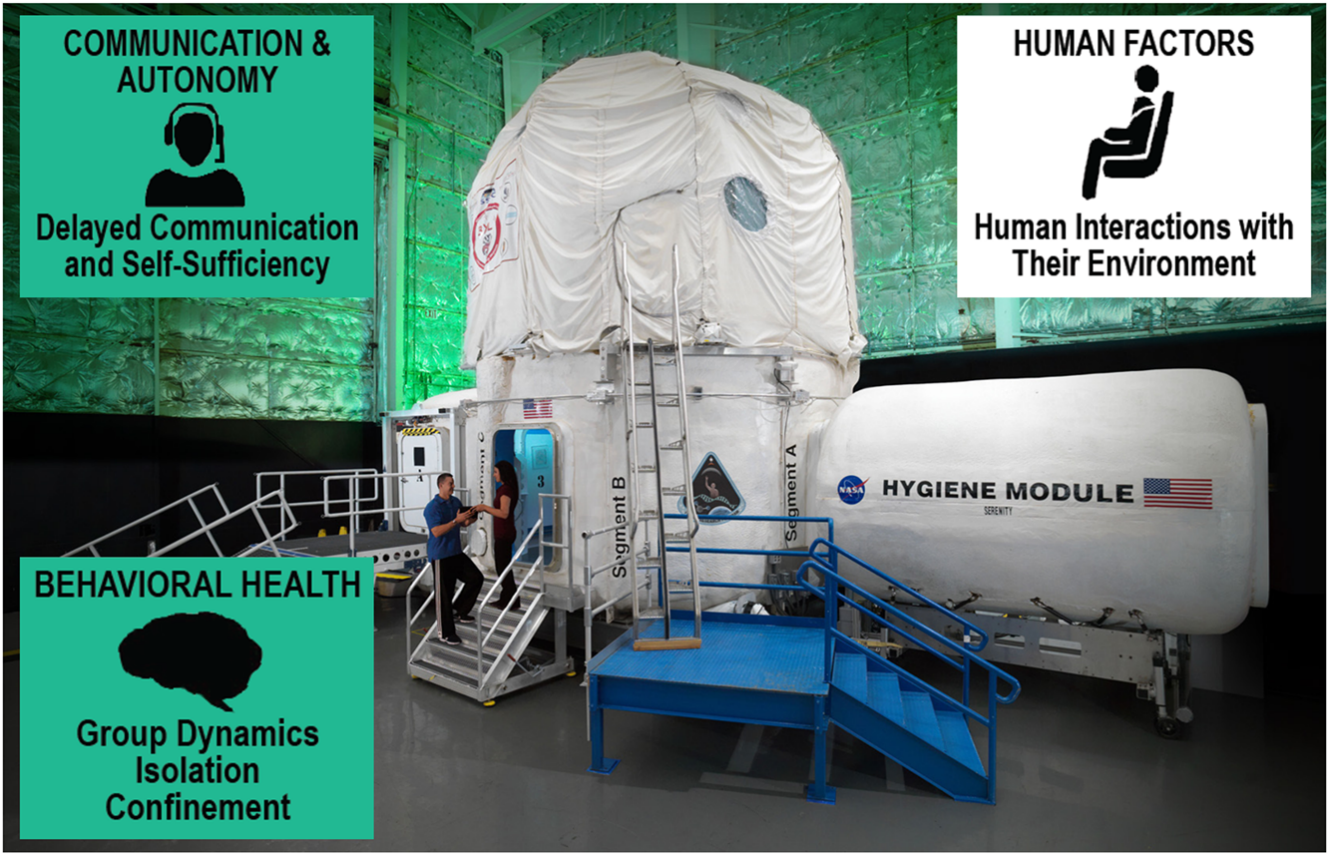
The NASA Human Research Exploration Analog. HERA is used to simulate environments and mission scenarios analogous to spaceflight to investigate a variety of behavioral and human factors issues. Images courtesy of NASA.

Future long-duration missions will pose threats to behavioral health and performance, such as extreme confinement in a small volume and communication delays, that are distinct from what is currently experienced on missions to the ISS. Analog research is concurrently underway to help further characterize the likelihood and consequence of an adverse behavioral outcome, and the effectiveness of potential countermeasures. Ground analogs, such as the Human Exploration Research Analog (HERA) at NASA Johnson Space Center, provide a test bed where controlled studies of small teams for periods up to 45 days, can be implemented (Fig. 10). HERA can be used to provide scenarios and environments analogous to space (e.g., isolation and confinement, communication delays, space food, and daily tasks and schedules) to investigate their effects on behavioral health, human factors, exploration medical capabilities, and communication and autonomy. Research in locations such as Antarctica also offer a unique opportunity to conduct research in less controlled but higher fidelity conditions. In general, these studies show an increased risk in deleterious effects such as decreased mood and increased stress, and in some instances, psychiatric outcomes (see the BMed Evidence Report).

In 2014, Basner and colleagues^62^ completed an assessment of crew health and performance in a 520-day mission at an isolation chamber in Moscow at the Institute for Biomedical Problems (IBMP). During this simulated mission to Mars, the crew of six completed behavioral questionnaires and additional testing weekly. One of six (20%) crew reported depressive symptoms based on the Beck Depression Inventory in 93% of mission weeks, which reached mild-to-moderate levels in >10% of mission weeks. Additional indications of changes in mood were observed via the Profile of Mood States. Additionally, two crewmembers who had the highest ratings of stress and physical exhaustion accounted for 85% of the perceived conflicts, and other crew demonstrated dysregulation in their circadian entrainment and sleep difficulties. Two of the six crewmembers reported no adverse behavioral symptoms during the missions^62^. Building on this work, the NASA HRP and the IBMP have ongoing studies in the SIRIUS project, a series of long-duration ground-analog missions for understanding the effects of isolation and confinement on human health and performance (http://www.nasa.gov/analogs/nek/about).

Finally, more recent research in the HERA analog at Johnson Space Center is underway to assess not only individual, psychiatric outcomes but also changes in team dynamics and team performance over time (Fig. 10). A recent publication reported that conceptual team performance (e.g., creativity) seems to decrease over time, while performance requiring cognitive function and coordinated action improved^66^. While results from additional team studies in HERA are currently under review, the Teams Risk Evidence Report (https://humanresearchroadmap.nasa.gov/Evidence/reports/Team.pdf) provides a thorough overview of the evidence surrounding team level outcomes.

In summary, evidence from spaceflight and spaceflight analogs suggests that the BMed Risk poses a high likelihood and high consequence risk for exploration. Given the possible synergistic effects of prolonged isolation and confinement, radiation exposure, and prolonged weightlessness, mitigating such enhanced risks faced by future crews are of highest priority to the NASA HRP.

## Inadequate food and nutrition

Historically, nutrition has driven the success—and often the failure—of terrestrial exploration missions. For space explorers, nutrition provides indispensable sustenance, provides potential countermeasures to some of the negative effects of space travel on human physiology, and also presents a multifaceted risk to the health and safety of astronauts (https://humanresearchroadmap.nasa.gov/Evidence/other/Nutrition-20150105.pdf).

At a minimum, the need to prevent nutrient deficiencies is absolute. This was proven on voyages during the Age of Sail, where scurvy—caused by vitamin C deficiency— killed more sailors than all other causes of death. On a closed (or even semi-closed) food system, the risk of nutrient deficiency is increased. On ISS missions, arriving vehicles typically bring some fresh fruits and/or vegetables to the crew. While limited in volume and shelf-life, these likely provide a valuable source of nutrients and phytochemicals every month or two. One underlying concern is that availability of these foods may be mitigating nutrition issues of the nominal food system, and without this external source of nutrients on exploration-class missions, those issues will be more likely to surface.

**Fig. 11:**
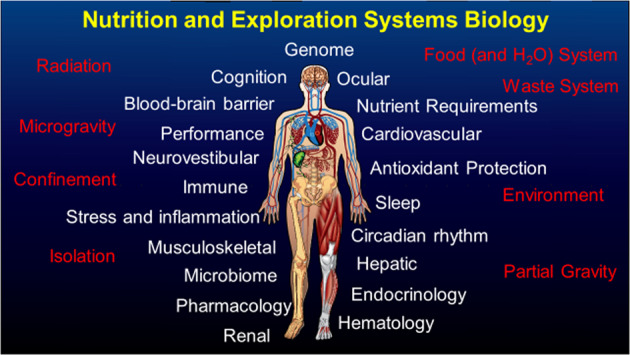
A depiction of the relationship of nutrition with exploration missions. Many of the physiological systems and performance characteristics that are touched by nutrition are shown in white text, while the unique elements of spacecraft and space exploration are shown in red text.

As a cross-cutting science, nutrition interfaces with many, if not all, physiological systems, along with many of the elements associated with space exploration, including the spacecraft environment (Fig. 11). Thus, beyond the basics of preventing deficiency of specific nutrients, at best, nutrition can serve as a countermeasure to mitigate risks to other systems. Conversely, at worst, diet and nutrition can exacerbate risks to other physiological systems and crew health. For example, many of the diseases of concern as related to space exploration are nutritionally modifiable on Earth, including cancer, cardiovascular disease, osteoporosis, sarcopenia, and cataracts.

The NASA Nutritional Biochemistry Laboratory approaches astronaut health with both operational and research efforts. These efforts aim to keep current crews healthy while working to understand and define optimal nutrition for future crews, to maximize performance and overall health while minimizing damaging effects of spaceflight exposure.

A Clinical Nutrition Assessment is conducted for ISS astronauts dating back to ISS Expedition 1^67,68^, which includes pre- and post-flight biochemical analyses conducted on blood and urine samples, along with in-flight monitoring of dietary intake and body mass. The biochemical assessments include a wide swath of nutritional indicators such as vitamins, minerals, proteins, hematology, bone markers, antioxidant markers, general chemistry, and renal stone risk. These data are reported to the flight surgeon soon after collection for use in the clinical care of the astronaut. Initial findings from the Clinical Nutritional Assessment protocol identified evidence of vitamin D deficiency, altered folate status, loss of body mass, increased kidney stone risk, and more^69,70^. These initial findings led to several research efforts (described below), including the Nutritional Status Assessment flight project, and research in the Antarctic on vitamin D supplementation^71,72^.

In addition to in-flight dietary intake monitoring, research to understand the impact and involvement of nutrition with other spaceflight risks such as bone loss and visual impairments, and interaction with exercise and spacecraft environment, are performed by the Nutrition Team using both flight and ground-analog research efforts. Tracking body mass is a very basic but nonetheless indispensable element of crew health^73^. Loss of body mass during spaceflight and in ground analogs of spaceflight is associated with exacerbated bone and muscle loss, cardiovascular degradation, increased oxidative stress, and more^70,73,74^. Historically, it was often assumed that some degree of body mass loss was to be expected, and that this was a typical part of adaptation to microgravity. Fluid loss is often assumed to be a key factor, but research has documented this to be a relatively small contributor, of approximately 1% of weight loss being fluid^74,75^. While on average, crewmembers on ISS missions have lost body mass over the course of flight, not all do^74^. Importantly, those that did not lose body mass managed to maintain bone mineral density (discussed below)^76^.

Bone loss has long been a concern for space travelers^77–81^. It has been shown that an increase in bone resorption was the likely culprit and that bone formation was largely unchanged in microgravity or ground analogs^77–79^. The search for a means to counteract this bone loss, and this hyper-resorptive state specifically, has been extensive. The potential for nutrition to mitigate this bone loss was identified early but studies of increasing intakes of calcium, or fluoride, or phosphate, were unsuccessful^74,77,79,82–84^.

Exercise provides a multisystem countermeasure, and heavy resistive exercise specifically provides for loading of bone to help mitigate weightlessness-induced bone loss.

**Fig. 12:**
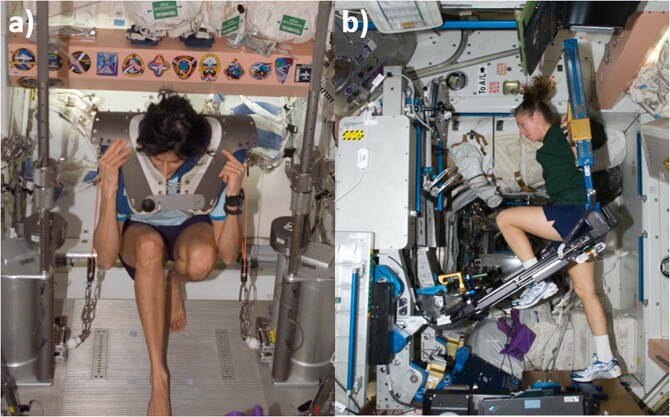
Resistance exercise devices on the ISS. Sunita Williams exercising on the iRED (**a**), and on a later mission, Sandy Magnus exercises on the much improved ARED device (**b**). Images courtesy of NASA.

In evaluating the data from astronauts using the first “interim” resistive exercise device (iRED) on ISS compared to a later, “advanced” resistive exercise device (ARED) (Fig. 12), it was quickly realized that exercise was not the only difference in these two groups of astronauts. ARED crews had better dietary intakes (as evidenced by maintenance of body mass) and better vitamin D status as a result of increased dose of supplementation and awareness of the importance of these supplements starting in 2006^76^. Bone mineral density was protected in these astronauts^76^, proving that diet and exercise are a powerful countermeasure combination. Follow-on evaluations showed similar results and further that the effects of microgravity exposure on bone health in men and women were similar^85^ despite differences in pre-flight bone mass.

From a purely nutrition perspective, ISS and associated ground analog research has identified several specific dietary effects on bone health. Fish intake, likely secondary to omega-3 fatty acid intake, is beneficial for bone health^86^. Conversely, high intakes of dietary protein^87,88^, iron^89^ and sodium^90^ are detrimental to bone. The mechanism of the effect of protein and sodium on bone are likely similar, with both contributing to the acidogenic potential of the diet, leading to bone dissolution^91,92^. This effect was recently documented in a diet and bone health study on ISS, where the acidogenic potential of the diet correlated with post-flight bone losses^93^. The data from terrestrial research, along with the more limited spaceflight research, clearly identifies nutrition as important in maintenance of bone health and in the mitigation of bone loss. While initial evaluations of dietary quality and health are underway at NASA, much work remains to document the full potential of nutrition to mitigate bone loss and other disease processes in space travelers.

Another health risk with nutrition underpinnings is SANS, which was described earlier. When this issue first arose, an examination of data from the aforementioned ISS Nutrition project was conducted. This analysis revealed that affected crewmembers had significantly higher circulating concentrations of homocysteine and other one-carbon pathway metabolites when compared to non-cases and that these differences existed *before* flight^53^. Many potential confounding factors were ruled out, including: sex, kidney function, vitamin status, and coffee consumption, among others. After identifying differences in one-carbon biochemistry, the next logical step was to examine the genetics—single-nucleotide polymorphisms (SNPs)—involved in this pathway as possible causes of the biochemical differences, but perhaps also their association with the astronaut ocular pathologies. An initial study examined a small set of SNPs—five to be exact—and when the data were statistically modeled, it was found that B-vitamin status and genetics were significant predictors of many of the observed ophthalmic outcomes in astronauts^94^. Interestingly, the same SNPs identified in astronauts to be associated with ophthalmic changes after flight were associated with greater changes in total retina thickness after a strict head-down tilt with 0.5% CO_2_ bed rest study^54^. A follow-on study is underway to evaluate a much broader look at one-carbon pathway and associated SNPs, potentially to help better characterize this relationship.

A hypothesis was developed to plausibly link these genetics and biochemical differences with these ophthalmic outcomes, as there is no existing literature regarding such a relationship. This multi-hit hypothesis posits that one-carbon pathway genetics is an indispensable factor, and that the combination with one or more other factors (e.g., fluid shifts, carbon dioxide, radiation, endocrine effects) lead to these pathologies. This has been detailed in a hypothesis paper^95^ and in a recent review^96^. In brief, the hypothesis is that genetics and B-vitamin status contribute to endothelial dysfunction, as folate (and other B-vitamins) play critical roles in nitric oxide synthesis and endothelial function. A disruption in nitric oxide synthesis can also lead to an activation of matrix metalloproteinase activation, increasing the turnover and breakdown of structural elements of the sclera, altering retinal elasticity and increasing susceptibility to fluid shifts to induce ophthalmic pathologies like optic disc edema and choroidal folds^54^. This is likely exacerbated cerebrally due to limitations of transport of B-vitamins across the blood-brain barrier. In or around the orbit, endothelial dysfunction, oxidative stress, and potentially individual anatomical differences contribute to leaky blood vessels, and subsequent edema. This can impinge on cerebrospinal fluid drainage from the head, increasing those fluid pressures, which can impinge upon the optic nerve and eye itself, yielding the aforementioned ophthalmic pathologies. These are hypotheses proposed as starting points for further research. Given the irrefutable biochemical and genetic findings to date, this research should be a high priority to either prove or dismiss these as contributing factors in SANS to mitigate that “red” risk.

Another intriguing element from this research is that there is a clinical population that has many of the same characteristics of affected astronauts (or characteristics that they are purported to have), and that is women with polycystic ovary syndrome (PCOS)^95,96^. Women with PCOS have higher circulating homocysteine concentrations (as do their siblings and fathers), and also have cardiovascular pathology, including endothelial dysfunction. Studies are underway between NASA and physicians at the Mayo Clinic in Minnesota to evaluate this further. If validated, women with PCOS might represent an analog population for astronaut ocular issues, and research to counteract this could benefit both populations^87^. This research may lead to the identification of one-carbon pathway genetic influences on cardiovascular function in astronauts (and women with PCOS). This information will *not* be used in any sort of selection process, for several reasons, but as a means to identify countermeasures. Given the effects are intertwined with vitamin status, and likely represent higher individual vitamin requirements, targeted B-vitamin supplementation is the most obvious, and lowest risk, countermeasure that needs to be tested. There is tremendous potential for nutrition research to solve one of the key risks to human health on space exploration missions.

To summarize, nutrition is a cross-cutting field that has influence on virtually every system in the body. While we need to understand nutrition to avoid frank deficiencies, we need to understand how optimizing nutrition might also help mitigate other spaceflight-induced human health risks. Examples of this are myriad, ranging from effects of dietary intake on cognition, performance, and morale, inadequate intake on cardiovascular performance, excess nutrient intakes, leading to excess storage and increased oxidative stress, nutrient insufficiencies, leading to bone loss, insufficient fruit and vegetable intake on bone health, radiation protection, and cardiovascular health, to name a just few. Throughout history, nutrition has served, or failed, many a journey to explore. We need to dare to use and expand our twenty-first century knowledge of nutrition, uniting medical and scientific teams, to enable future exploration beyond LEO, while simultaneously benefitting humanity.

## Summary

The NASA Human Research Program is focused on developing the tools and technologies needed to control the high priority “red” risks to an acceptable level—a great challenge as the risks do not exist in the vacuum of space as standalone entities. They are inherently interconnected and represent the intersection points where the five hazards of spaceflight overlap, and nature meets nurture. This is the space exposome: the total sum of spaceflight and lifetime exposures and how they relate to individual genetics and determine the whole-body outcome. The space exposome will be an important unifying concept as the hazards and risks of spaceflight are evaluated in a systems biology framework to fully uncover the emergent effects of the extraterrestrial experience on the human body. This framework will provide a path forward for mitigating detrimental health and performance outcomes that may stand in the way of successful, long-duration space travel, especially as NASA plans for a return to the Moon, to stay, and beyond to Mars.
